# MicroRNA Regulation and its Biological Significance in Personalized Medicine and Aging

**DOI:** 10.2174/138920209788185216

**Published:** 2009-05

**Authors:** Dr. Eugenia Wang

The recent explosion of research on the role of noncoding RNAs (ncRNA) in the control of gene expression has revealed multifaceted implications concerning the regulation of numerous mammalian systems, ranging from determination of cell fate during development to maintenance of terminally differentiated states. Among the various types of ncRNA, microRNAs are probably the best known group; their functional impact in signaling pathways controls normal processes regulating apoptosis, cell cycle traverse, differentiation, cytoskeletal organization, *etc*. at the post-transcriptional level, by either degrading their target genes’ messages through binding at the coding region, or inhibiting translation at the 3’-untranslated region. Not surprisingly, dysregulation of microRNA expression has been linked to the pathogenesis of a variety of diseases, among which cancer, cardiovascular disorders, and neurodegeneration have proven fertile grounds for investigation.

This issue focuses on a discussion of microRNA’s post-transcriptional control of the aging process. The paper by Ibáñez-Ventoso and Driscoll provides a comprehensive review of the potential impact of microRNA expression on health span, based upon the well-known aging model, *C. elegans*, with the latest miRbase 10.1 version stating that 73 out of 139 worm miRNAs have sequence relationship to known human miRNAs. Understanding how they control the signaling networks that modulate aging in the worm should provide major insights into the aging process of mammalian species, including man. Notwithstanding the essential role of microRNA in cancer etiology, the biggest impact of microRNA regulation of post-transcriptional control of gene expression may be that associated with neurodegeneration. The paper by Maes, *et al*. reviews the basic knowledge of how microRNAs control neuronal cell fate during development, and includes recent evidence that changes in expression levels of this noncoding RNA species are vital to the pathogenesis of a wide variety of disorders associated with the central nervous system, as well as being systemically manifested in peripheral blood mononuclear cells. Of all the complex hormonal control mechanisms of the mammalian aging process, estrogen is perhaps the most significant single factor, since many of the disorders seen in the female aging population are attributed to the functional decline associated with menopause. In this context, the paper by Klinge describes aberrant patterns of microRNA expression in assorted estrogen-related cancers, the most notorious being breast cancer; the role of estrogen-regulated changes in expression of microRNAs and their downstream target genes in the aging process are discussed. Beyond the recognition of microRNAs as a vital molecular species for repression of gene expression at the post-transcriptional level, increasing reports indicate that their transcriptional regulation is equally important, and follows the well-established mechanisms of promoter-dependent regulation with coordinated genomic organization in terms of transcriptional start sites, *cis* elements, activation by transcriptional factors, *etc*. Along this line, Liang, * et al*. reviews the genomic structures and organization of microRNA genes, and how changes such as oxidative stress during aging can affect the transcriptional regulation of microRNA expression. Among all the transcriptional factors affecting microRNA expression, p53 may be the best known, since its multiple functions are implicated in cancer, senescence, and apoptosis. In the paper by Takwi and Li, the p53-directed pathway is reviewed in detail, specifically how it activates microRNAs which affect genes involved in various components of this noted signaling network. In conclusion, this issue provides, for the first time, a collection of papers describing the importance of microRNAs in controlling the aging process, and their possible roles in the etiology of age-dependent diseases. We hope this compendium will stimulate further research in this area, a fertile ground for study of the post-transcriptional control of gene expression during the aging process.

